# VEGF and VEGF type C play an important role in angiogenesis and lymphangiogenesis in human malignant mesothelioma tumours

**DOI:** 10.1038/sj.bjc.6690650

**Published:** 1999-09

**Authors:** Y Ohta, V Shridhar, R K Bright, G P Kalemkerian, W Du, M Carbone, Y Watanabe, H I Pass

**Affiliations:** Aerodigestive Program and; Hematology-Oncology, Barbara Ann Karmanos Cancer Institute, Detroit, MI, USA; Loyola Cancer Center, Maywood, IL, USA; First Department of Surgery School of Medicine, Kanazawa University, Kanazawa, Japan

**Keywords:** vascular endothelial growth factor, VEGF, VEGF-C, angiogenesis, lymphangiogenesis, malignant mesothelioma

## Abstract

The vascular endothelial growth factor (VEGF) family is a novel regulator of endothelial cell proliferation. We assessed the mRNA expression of VEGF, VEGF type C (VEGF-C) and their receptors together with the microvessel density (VD) and microlymphatic vessel density (LVD) in pursuit of their connection and prognostic value in malignant pleural mesothelioma (MPM). We used four human MPM cell lines, 54 MPM tumours and five normal pleural tissues. Expression levels for receptors and ligands were assessed by semiquantitative reverse transcriptase polymerase chain reaction analysis. Microvessels were highlighted by immunohistochemical staining for factor VIII. The discrimination of lymphatics was performed by enzyme-histochemistry for 5′-nucleotidase after adequate inhibition of non-specific activity. The expression levels of VEGF, VEGF-C and VEGFRs were high in all MPM cell lines. The percentages of tumours with higher expression compared to the mean values of normal pleural tissues were 31.5% (17/54) for VEGF, 66.7% (36/54) for VEGF-C, 20.4% (11/54) for fms-like tyrosine kinase (flt)-1, 42.6% (23/54) for kinase insert domain-containing recepter (KDR) and 59.3% (32/54) for flt-4. Significant positive correlations were found between VEGF-C and flt-4, VEGF and KDR, VEGF and flt-1 in tumour tissues. The association between LVD and VEGF-C expression level was especially strong (*P* < 0.0001, *r* = 0.63). There were also significant correlations between LVD and flt-4, and VD and VEGF. No correlation, however, was found between LVD and nodal metastasis. VD was a negative prognostic indicator in this study. The associations between VEGF/VEGF-C and vessel density suggest that these factors play an important role in angiogenesis and lymphangiogenesis in this tumour, and assessment of vascularity may be a useful prognostic indicator for MPM patients. © 1999 Cancer Research Campaign


*British Journal of Cancer* (1999) **81**(1), 54–61
© 1999 Cancer Research Campaign
Article no. bjoc.1999.0650

### VEGF and VEGF type C play an important role in angiogenesis and lymphangiogenesis in human malignant mesothelioma tumours

**Y Ohta[1,4], V Shridhar[1], RK Bright[1], GP Kalemkerian[1], W Du[2], M Carbone[3], Y Watanabe[4] and HI Pass[1]**

[1]Aerodigestive Program and [2]Hematology-Oncology, Barbara Ann Karmanos Cancer Institute, Wayne State University School of Medicine, Detroit, MI, USA; [3]Loyola Cancer Center, Maywood, IL, USA; [4]First Department of Surgery, School of Medicine, Kanazawa University, Kanazawa, Japan

**Summary** The vascular endothelial growth factor (VEGF) family is a novel regulator of endothelial cell proliferation. We assessed the mRNA expression of VEGF, VEGF type C (VEGF-C) and their receptors together with the microvessel density (VD) and microlymphatic vessel density (LVD) in pursuit of their connection and prognostic value in malignant pleural mesothelioma (MPM). We used four human MPM cell lines, 54 MPM tumours and five normal pleural tissues. Expression levels for receptors and ligands were assessed by semiquantitative reverse transcriptase polymerase chain reaction analysis. Microvessels were highlighted by immunohistochemical staining for factor VIII. The discrimination of lymphatics was performed by enzyme-histochemistry for 5′-nucleotidase after adequate inhibition of non-specific activity. The expression levels of VEGF, VEGF-C and VEGFRs were high in all MPM cell lines. The percentages of tumours with higher expression compared to the mean values of normal pleural tissues were 31.5% (17/54) for VEGF, 66.7% (36/54) for VEGF-C, 20.4% (11/54) for fms-like tyrosine kinase (flt)-1, 42.6% (23/54) for kinase insert domain-containing recepter (KDR) and 59.3% (32/54) for flt-4. Significant positive correlations were found between VEGF-C and flt-4, VEGF and KDR, VEGF and flt-1 in tumour tissues. The association between LVD and VEGF-C expression level was especially strong ($P < 0.0001$, $r = 0.63$). There were also significant correlations between LVD and flt-4, and VD and VEGF. No correlation, however, was found between LVD and nodal metastasis. VD was a negative prognostic indicator in this study. The associations between VEGF/VEGF-C and vessel density suggest that these factors play an important role in angiogenesis and lymphangiogenesis in this tumour, and assessment of vascularity may be a useful prognostic indicator for MPM patients.

**Keywords:** vascular endothelial growth factor; VEGF; VEGF-C; angiogenesis; lymphangiogenesis; malignant mesothelioma

Asbestos had been widely used for centuries before a causal relationship between malignant pleural mesothelioma (MPM) and exposure to asbestos was confirmed. Although the use of asbestos has recently been restricted, the incidence of MPM remains high and is steadily increasing due to the long latent period for MPM after the exposure to asbestos (Mossman et al, 1996). Advanced MPM remains an aggressive and highly lethal disease due to its marked resistance to conventional treatments, including surgical resection, chemotherapy and radiotherapy.

Angiogenesis is crucial for the proliferation of tumour cells, and anti-angiogenic therapy is a promising strategy aimed at inhibiting tumour growth, invasion and metastasis (Folkman et al, 1995). Among the many reported angiogenic factors, vascular endothelial growth factor (VEGF) is the most powerful endothelial cell-specific mitogen associated with tumour neovascularization. While a number of investigators have reported the direct relationship between microvessel density (VD) and VEGF expression within a variety of tumours (Toi et al, 1994; Mattern et al, 1995; Samoto et al, 1995; Takahashi et al, 1995), recent studies also suggest that VEGF type B, C, D and E are novel regulators of endothelial cell proliferation (Grimmond et al, 1996; Joukov et al, 1996; Lee et al, 1996; Olofsson et al, 1996; Yamada et al, 1997; Meyer et al, 1999). Interestingly, the function of VEGF-C appears to extend to the lymphatic system where it serves as a ligand for fms-like tyrosine kinase 4 (flt-4) (Kukk et al, 1996; Jeltsch et al, 1997). However, lymphangiogenesis within tumours has not been documented.

For MPM, little information on tumour angiogenesis is available. In this study, we assessed the expression of VEGF, VEGF-C and VEGFRs (KDR, flt-1 and flt-4) in human MPM cell lines, MPM tumour samples and normal pleural tissue. We used the reverse transcriptase polymerase chain reaction technique (RT-PCR) to assess the expression of each factor, and we also assessed the VD and microlymphatic vessel density (LVD) within tumours using immunohistochemistry for factor VIII and enzyme-histochemistry for 5′-nucleotidase (5′-NA) respectively. The expression of the associated angiogenic factors and receptors was also determined. Clinical correlations of angiogenesis with survival from treatments for the patients was also performed.

### MATERIALS AND METHODS

### Cell lines and tissue samples

The human malignant mesothelioma cell lines (H-meso, H2818, H2591 and H2595) used in this study were all developed from tumours diagnosed using a well-defined panel of immunohistochemical markers (Pass et al, 1995). H-meso is commercially available (Biomeasure, Hopkinton, MA, USA), while H2818,

*Received 28 October 1998*
*Accepted 16 February 1999*

*Correspondence to:* Y Ohta, First Department of Surgery, Kanazawa University, School of Medicine, Kanazawa 920-8641, Japan

**Table 1** Nucleotide sequences of the primers used and their PCR conditions.

| Angiogenic factors | Primer sets | PCR cycle | Product size (bp) |
|---|---|---|---|
| VEGF | 5′-GAAGTGGTGAAGTTCATGGATGTC-3′ (sense)<br>5′-CGATCGTTCTGTATCAGTCTTTCC-3′ (antisense) | 30 | 408 (VEGF121)<br>541 (VEGF165)<br>613 (VEGF185) |
| VEGF-C | 5′-CATGTACGAACCGCCAG-3′ (sense)<br>5′-TTGGCTGTTTGGTCATTGGC-3′ (antisense) | 25 | 320 |
| Flt-1 | 5′-GAGAATTCACTATGGAAGATCTGATTTCTTACAGT-3′ (sense)<br>5′-GAGCATGCGGTAAAATACACATGTGCTTCTAG-3′ (antisense) | 30 | 1098 |
| Flt-4 | 5′-AGCCATTCATCAACAAGCCT-3′ (sense)<br>5′-GGCAACAGCTGGATGTCATA-3′ (antisense) | 25 | 298 |
| KDR | 5′-TATAGATGGTGTAACCCGGA-3′ (sense)<br>5′-TTTGTCACTGAGACAGCTTGG-3′ (antisense) | 30 | 555 |

H2591 and H2595 were developed from resected MPM tumours at the National Cancer Institute (Bethesda, MD, USA) (Pass et al, 1995). A human fibrosarcoma cell line (HT1080) was used as a positive control for VEGF, VEGF-C, flt-1 and flt-4, while HUVEC was used for kinase insert domain-containing receptor (KDR). Cell lines were maintained in RPMI-1640 media (Gibco-BRL, Gaithersburg, MD, USA) supplemented with 10% fetal bovine serum (FBS), 2 mM L-glutamine, 10 mM HEPES buffer, 500 units $ml^{-1}$ penicillin and 500 units $ml^{-1}$ streptomycin at 37°C under a humidified atmosphere of 5% carbon dioxide. Five normal pleural tissue samples were obtained from patients with non-cancerous disease (either for repair of hiatus hernia or diagnosis of pulmonary infiltrates). In all these cases, the resected pleural tissue was confirmed to be free of malignancy by pathological examination. And 54 malignant mesothelioma tumours were randomly chosen from a bank of snap-frozen samples collected by one of us (HIP). For tumour samples, consecutive 6-μm cryosections were cut from the invasive tumour margin for immunohistochemical and enzyme-histochemical studies. The pathologic classification of each sample was confirmed by review of haematoxylin and eosin (H&E)-stained sections. The pathological MPM subtypes identified were: epithelial (E), 44; sarcomatous (S), 4; and mixed (M), 6. According to the new international TNM staging system (Rusch, 1996), 50 cases could be classified based on pathological findings [stage II in nine (E, 7; S, 2), III in 36 (E, 28; S, 2; M, 6) and IV in five (E, 4; M, 1)].

### RT-PCR analysis

Total RNA was extracted from the human mesothelioma cell lines, control cell lines (HT1080 and HUVEC), normal pleural tissues, and each resected MPM tumour using TRIzol (Life Technologies, Inc., Grand Island, NY, USA) according to the standard acid–guanidium–phenol–chloroform method. Total RNA (1 μg) was denatured together with oligo-dT primer (10 pmol) for 15 min at 68°C. After this was chilled on ice for 5 min, poly-adenosine (poly-A) RNA was reverse-transcribed at 42°C for 90 min in RT solution (50 mM Tris–HCl (hychochloric acid), pH 8.3; 40 mM potassium chloride (KCl); 8 mM magnesium chloride ($MgCl_2$); 0.5 mM each dNTP; 225 μg $ml^{-1}$ bovine serum albumin; 5 mM dithiothreitol; 20 units RNasin (Life Technologies, Inc., Grand Island, NY, USA) and 4 units AMV reverse transcriptase (Gibco-BRL, Gaithersburg, MD, USA)). The cDNA was incubated at 95°C for 5 min to inactivate the reverse transcriptase, and served as the template for PCR amplification. For quantitative evaluation of the amplified product, PCR encompassing 20–40 cycles was preliminarily performed to determine the most suitable number of amplifications for each factor. PCR was performed after adding 80 μl of PCR mixture (50 mM Tris–HCl, pH 8.3; 40 mM KCl; 8 mM $MgCl_2$; 0.5 mM each dNTP; 50 pmol of each the sense and the antisense primer; 2.5 units of *Taq* polymerase (Takara, Kyoto, Japan)). Each cycle consisted of: 1 min at 94°C, 1 min at 58°C and 2 min at 72°C for VEGF, flt-1 and KDR; and 1 min at 94°C, 1 min at 52°C and 1.5 min at 72°C for VEGF type C, flt-4 and β-actin. PCR primers used and PCR conditions are shown in Table 1. The PCR products were electrophoresed through a 1.0% agarose gel (4-mm thick). The gel was fluorescently stained in SYBR Green I stain Solution (FMC Bio-Products, Rockland, ME, USA) at a 1:10 000 dilution in 1 × TAE buffer (pH 7.2) for 30 min. The intensity of the PCR product bands was measured by Storm™ image analyser (Molecular Dynamics, Inc., Sunnyvale, CA, USA). Each expression was standardized using the β-actin signal as an internal control and a densitometry index defined. For negative control of each factor, PCR was done excepting the template cDNA.

### Enzyme-histochemistry

An enzyme-histochemical reaction for 5′-NA was done according to the leading method of Wachstein et al (1954). Briefly, cryostat sections were fixed with 5% paraformaldehyde for 10–60 min (10, 20, 30, 40, 50 and 60 min for each sample). This step-wise fixation was carried out in order to discover the appropriate conditions to maximally exhibit 5′-NA activity in microlymphatic vessels compared to that in blood vessels. Next, after washing with water for 5 min, sections were treated with an enzyme reaction for 2 h at 37°C using a substrate mixture (20 ml of 0.2 M Tris–maleate buffer (pH 7.2), 25 mg adenosine-5-monophosphate (Sigma, St Louis, MO, USA), 20 mg tetramisole (Sigma, St Louis, MO, USA), 3.5 mg sucrose, 5 ml 0.1 M magnesium sulphate, 3 ml of 2% $Pb(NO_3)_2$, 20 ml water). After the sections were washed with water for 15 min, they were reacted with a 1% ammonium sulphide solution (Sigma, St Louis, MO, USA) for 2 min. The sections were counterstained with methyl green and mounted using glycerol (Sigma, St Louis, MO, USA).

### Immunohistochemistry

Consecutive cryostat sections were used for the immunohistochemical assessment of VD. The primary antibodies used in this study

*© 1999 Cancer Research Campaign* 

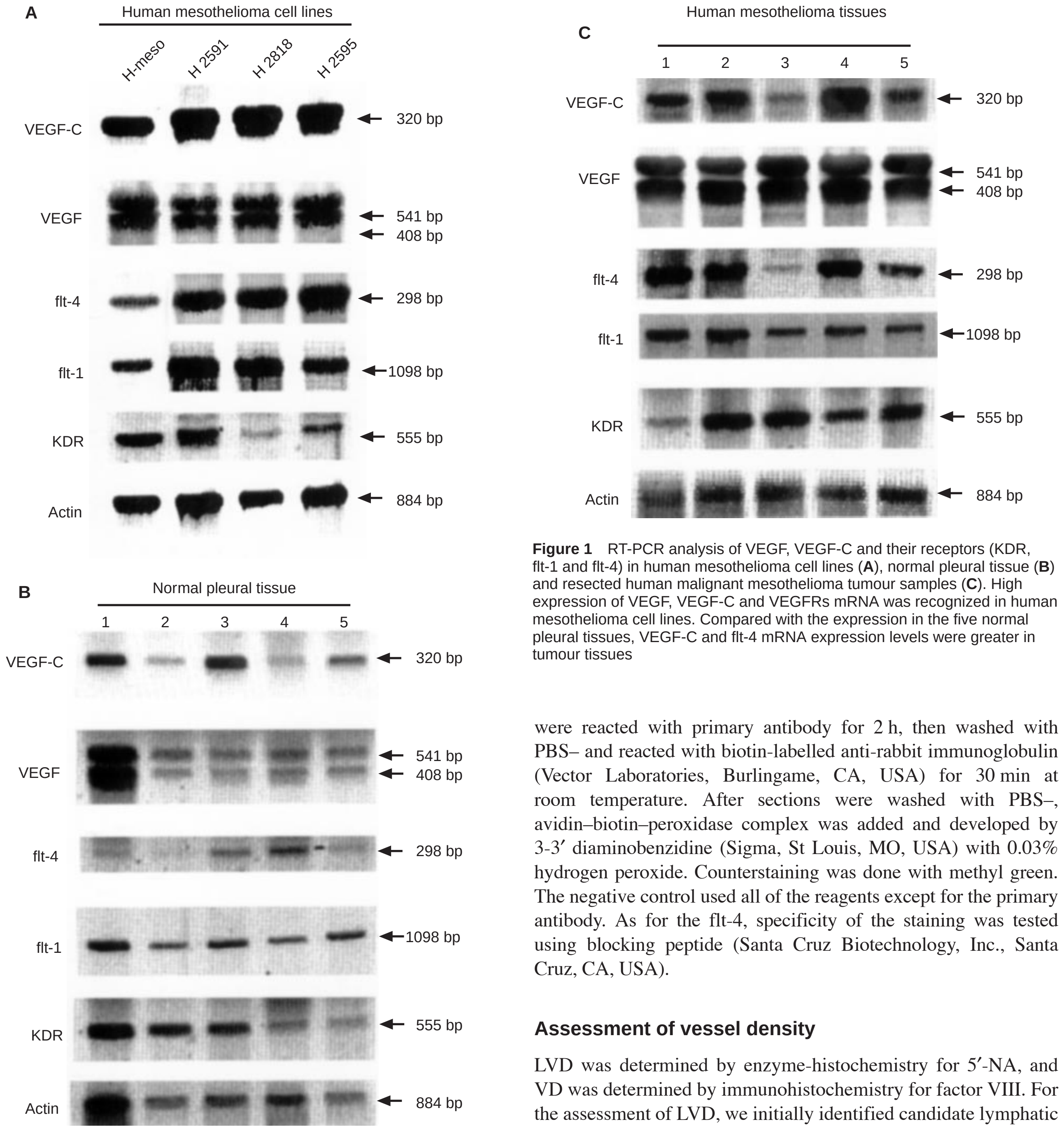

**Figure 1** RT-PCR analysis of VEGF, VEGF-C and their receptors (KDR, flt-1 and flt-4) in human mesothelioma cell lines (**A**), normal pleural tissue (**B**) and resected human malignant mesothelioma tumour samples (**C**). High expression of VEGF, VEGF-C and VEGFRs mRNA was recognized in human mesothelioma cell lines. Compared with the expression in the five normal pleural tissues, VEGF-C and flt-4 mRNA expression levels were greater in tumour tissues were an anti-factor VIII rabbit polyclonal antibody at a 1:200 dilution (Dako Corp., Carpinteria, CA, USA) and an anti-flt-4 rabbit polyclonal antibody at a 1:200 dilution (Santa Cruz Biotechnology, Inc., Santa Cruz, CA, USA). The staining was done by the immunoperoxidase technique. After air-drying for 10 min sections were fixed with acetone for 10 min at –20°C. Then the cryostat sections were washed in Dulbecco's phosphate-buffered saline (pH 7.2) without calcium or magnesium (PBS–) and endogenous peroxidase was blocked by treatment with 0.3% hydrogen peroxide in methanol for 30 min. After washing with PBS–, the sections were blocked with normal goat serum diluted tenfold with PBS– for 20 min at room temperature. After washing with PBS–, the sections were reacted with primary antibody for 2 h, then washed with PBS– and reacted with biotin-labelled anti-rabbit immunoglobulin (Vector Laboratories, Burlingame, CA, USA) for 30 min at room temperature. After sections were washed with PBS–, avidin–biotin–peroxidase complex was added and developed by 3-3′ diaminobenzidine (Sigma, St Louis, MO, USA) with 0.03% hydrogen peroxide. Counterstaining was done with methyl green. The negative control used all of the reagents except for the primary antibody. As for the flt-4, specificity of the staining was tested using blocking peptide (Santa Cruz Biotechnology, Inc., Santa Cruz, CA, USA).

### Assessment of vessel density

LVD was determined by enzyme-histochemistry for 5′-NA, and VD was determined by immunohistochemistry for factor VIII. For the assessment of LVD, we initially identified candidate lymphatic vessels and blood vessels in consecutive H&E-stained and immunohistochemically stained sections for each sample. Then, we chose the most appropriate enzyme-histochemical stained section among the six sections with different fixation times for each sample, i.e. the slides with maximal 5′-NA activity in lymphatic vessels compared to in blood vessels. The vessel densities were assessed blindly by two investigators according to the method previously described by Weidner et al (1991). After the areas of the highest vascularization were chosen under low power (100 × magnification), vessel counts within tumours were done in the three fields at 400 × magnification for VD and at 200 × magnification for LVD. The average counts of the three fields were recorded and the mean of the two investigators findings was used for the final VD.

© 1999 Cancer Research Campaign

**Table 2** Expression of VEGF, VEGF-C and VEGFRs mRNA in malignant mesothelioma tumours

| | Percentage of positive tumours (cases) | [a]Percentage of overexpression (cases) |
|---|---|---|
| VEGF | 75.9 (41/54) | 31.5 (17/54) |
| VEGF-C | 85.2 (46/54) | 66.7 (36/54) |
| flt-1 | 74.1 (40/54) | 20.4 (11/54) |
| KDR | 90.7 (49/54) | 42.6 (23/54) |
| flt-4 | 96.3 (52/54) | 59.3 (32/54) |

[a]> each mean value of normal pleural tissue.

**Table 3** The mean densitometry index of each factor in MPM tumours and normal pleural tissue samples

| | MPM tumours (*n* = 54) | Normal pleural tissue (*n* = 5) |
|---|---|---|
| VEGF-C | 0.87 ± 0.15 | 0.16 ± 0.03 |
| flt-4 | 1.24 ± 0.25 | 0.34 ± 0.07 |
| KDR | 1.73 ± 0.38 | 1.08 ± 0.23 |
| VEGF | 1.80 ± 0.41 | 1.80 ± 0.60 |
| flt-1 | 0.50 ± 0.11 | 0.95 ± 0.46 |

### Statistics

Differences in densitometry indices were analysed using the Mann–Whitney *U*-test. The Spearman's rank correlation coefficient test was used to examine the associations between different variables. To assess the prognostic effect on overall survival, VEGF, VEGF-C, VEGFRs, VD and LVD were classified as high or low expressing group relative to the mean values of densitometry indices in resected tumours. Two combination factors, COM-VEGF and COM-VEGF-C, were defined to reduce the dimensionality of the independent variables list. The COM-VEGF factor was defined as 'positive' if two or more of the three associated variables (VEGF, flt-1, KDR) were strongly expressed, 'negative' if otherwise. The COM-VEGF-C factor was defined as 'positive' if both VEGF-C and flt-4 were strongly expressed, 'negative' if otherwise. Survival curves were obtained by the Kaplan–Meier method, and compared univariately by log-rank tests. The effects of age (over vs under 50 years), gender, pathological stage (I, II, vs III, IV), pathological subtype (epithelial vs others), nodal status (positive vs negative), VEGF, VEGF-C, VEGFRs, VD and LVD on overall survival were assessed multivariately using the Cox proportional hazard regression along with a step-wise procedure for variable selection. Factors with *P*-value < 0.10 were included in the final model.

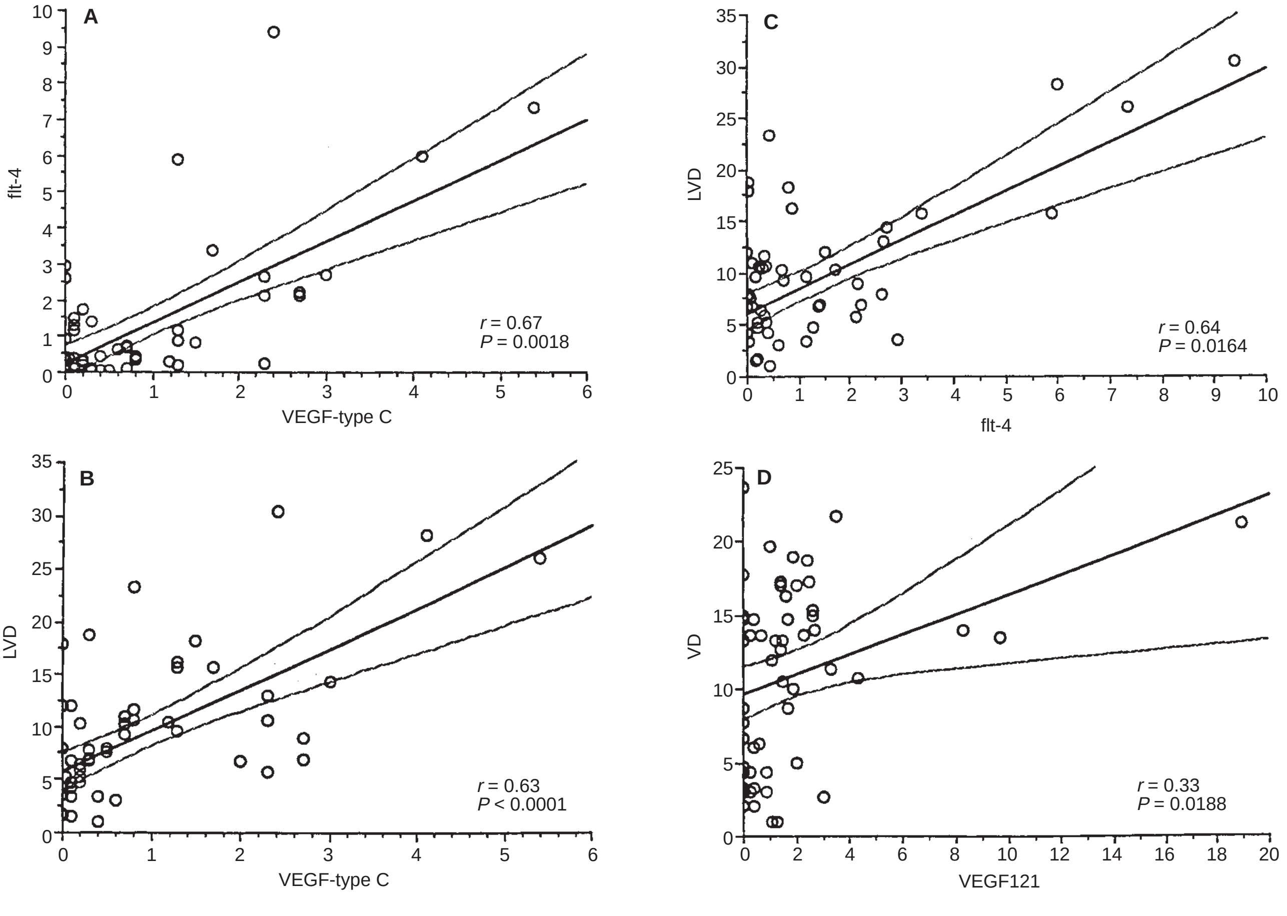

**Figure 2** Correlation (non-parametric) between VEGF-C and flt-4 mRNA expression (**A**), VEGF-C mRNA expression and LVD (**B**), flt-4 mRNA expression and LVD (**C**) and VEGF mRNA expression and VD (**D**) within MPM tumours

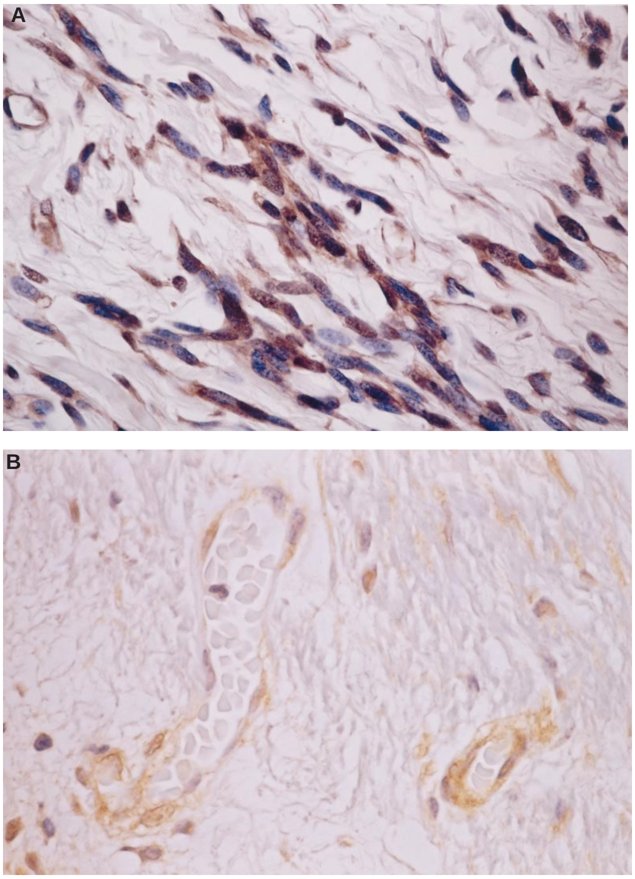

**Figure 3** (**A**) Staining of flt-4 using polyclonal antibody in malignant pleural mesothelioma tissue. Cytoplasmic staining was positive in tumour cells. (**B**) Weakly staining of flt-4 was partly identified in vascular endothelial cells

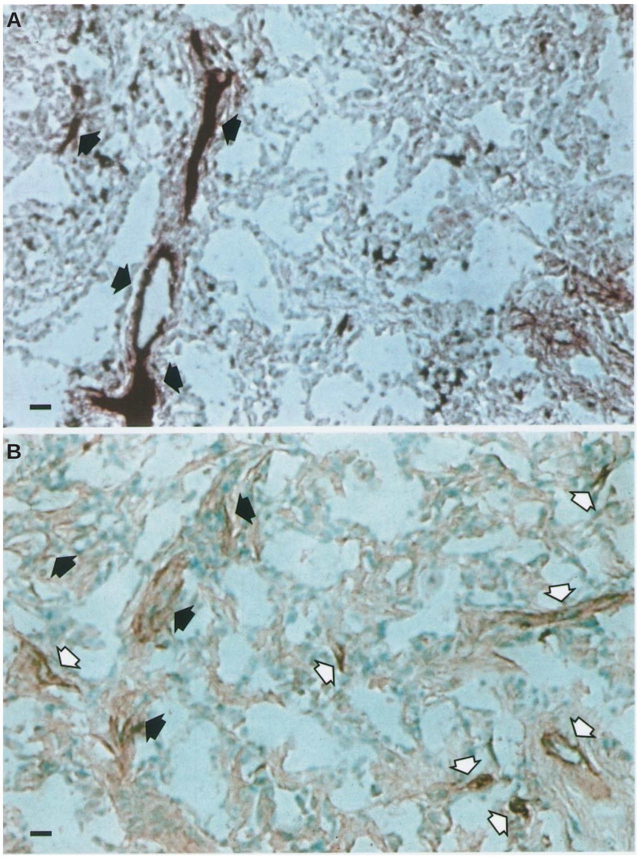

**Figure 4** 5′-nucleotidase reaction of microvessels within tumours by enzyme-histochemistry for 5′-NA (**A**) and immunohistochemical staining for factor VIII antigen (**B**) of the consecutive cryostat sections. Theoretically, closed arrows show lymphatic vessels and open arrows show blood vessels. Closed arrows in **A** were counted for LVD and both closed and open arrows in **B** were counted for VD in this study. Scale bar, 40 μm

### RESULTS

### Expression of VEGF, VEGF-C and VEGFRs in normal pleural tissues, human mesothelioma cell lines and resected mesothelioma tumours

High VEGF, VEGF-C and VEGFRs (KDR, flt-1 and flt-4) mRNA expression was noted in all human MPM cell lines (Figure 1A). In MPM tumours, the percentages of positive tumours were, VEGF, 75.9% (41/54 cases); VEGF-C, 85.2% (46/54); flt-1, 74.1% (40/54); KDR, 90.7% (49/54); flt-4, 96.3% (52/54). The percentages of tumours with higher expression compared to the mean values of normal pleural tissues were 31.5% (17/54) for VEGF, 66.7% (36/54) for VEGF-C, 20.4% (11/54) for flt-1, 42.6% (23/54) for KDR and 59.3% (32/54) for flt-4 (Table 2). Compared to the mean values in resected tumours, higher densitometry index of VEGF was found in two (case nos 2 and 5 in Figure 1B) and higher index of VEGF-C was also recognized in two out of five normal pleural samples (case nos 3 and 5 in Figure 1B). The sample data of resected tumour specimens are shown in Figure 1C. Compared with normal pleural tissues, the mean densitometry indexes of VEGF-C (0.87 ± 0.15 vs 0.16 ± 0.03) (± s.e.m.), flt-4 (1.24 ± 0.25 vs 0.34 ± 0.07) and KDR (1.73 ± 0.38 vs 1.08 ± 0.23) tended to be greater in MPM tumours (Table 3). Positive correlations were observed between VEGF-C and flt-4 ($P = 0.0018$, $r = 0.67$, Figure 2A), VEGF and KDR ($P = 0.0002$, $r = 0.39$), and VEGF and flt-1 ($P = 0.0089$, $r = 0.27$).

Immunohistochemically, flt-4 antigen was identified in the cytoplasm of malignant pleural mesothelioma cells (Figure 3A) and partly in vascular endothelial cells (Figure 3B).

### LVD and VD within MPM tumour tissues

The 5′-NA staining was limited to vessels in 31 of the 54 specimens (57.4%). In these samples, fixation for 30–40 min was optimal for highlighting 5′-NA activity in lymphatic vessels while suppressing 5′-NA activity in blood vessels (Figure 4). In 23 cases (42.6%), 5′-NA staining was seen both in vessels and in tumour cells or stromal elements. In 20 of these 23 cases, the intensity of this non-specific staining was very low compared to that of the lymphatics, and assessment of LVD was easily achieved. However, in three cases, the intensity of non-specific staining remained very high despite 60-min of fixation. In these three cases, LVD was assessed by avoiding areas with high non-specific activity. The mean VD and LVD were 10.9 ± 0.8 (range 0–31) and 9.1 ± 0.9 (range 1–24) respectively. LVD and VEGF-C mRNA expression were strongly associated ($P < 0.0001$, $r = 0.63$, Figure 2B). There were also significant positive relationships between LVD and flt-4 expression ($P = 0.0164$, $r = 0.64$, Figure 2C), and VD and VEGF ($P = 0.0188$, $r = 0.33$, Figure 2D).

© 1999 Cancer Research Campaign

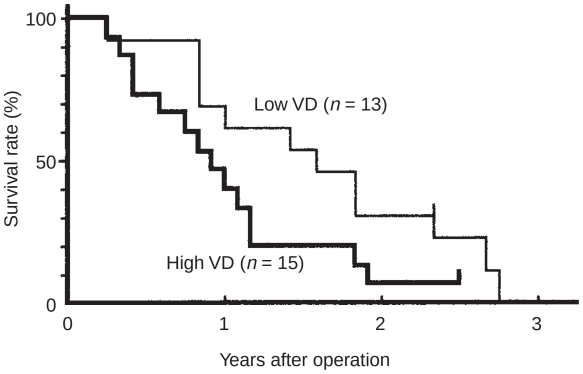

**Figure 5** Kaplan–Meier survival plots in stage III patients with epithelial type MPM who underwent standardized resections (*n* = 28). A tumour was included in the high VD group if VD was greater than 11. High VD tended to be associated with poor survival (*P* = 0.0866)

**Table 4** Cox proportional hazard regression analysis using 46 malignant pleural mesothelioma patients with standardized resections

| | Hazard ratio | *P* | 95% CI |
|---|---|---|---|
| Gender | 3.437 | 0.0067 | 1.409 – 8.384 |
| Stage (I, II vs III, IV) | 3.729 | 0.0091 | 1.387 – 10.025 |
| VD | 2.247 | 0.0326 | 1.070 – 4.719 |
| *COM-VEGF[a] | 0.400 | 0.0731 | 0.147 – 1.089 |

[a]A combination factor of VEGF, flt-1, and KDR. It was defined as 'positive' if two or more of the three associated variables are strongly expressed.

### Association with clinicopathological findings

Histopathological information on lymph node metastasis was available in 47 cases. The mean LVD and VD were 8.3 ± 1.0 and 10.9 ± 1.1 in node-positive cases (*n* = 30), and 10.2 ± 1.4 and 11.0 ± 1.5 in node-negative cases (*n* = 17) respectively. Nodal status had no relationship with LVD (*P* = 0.1351) or VD (*P* = 0.8856). VEGF and VEGF-C mRNA expression levels were 1.9 ± 0.6 and 0.8 ± 0.2, respectively, in node-positive cases, and 1.3 ± 0.2 and 0.9 ± 0.2, respectively, in node-negative cases. The differences were not statistically significant. In stage III patients with epithelial type who underwent standardized resections (*n* = 28), the median survival of high and low VD groups were 11 and 17 months respectively. The 1-year survival rates for high and low VD were 40.0% and 61.5% respectively. In this analysis, high VD tended to correlate with poor survival (*P* = 0.0866, Figure 5). Using all patients with standardized resections (*n* = 46), age, nodal status, histological subtype, VEGF-C, flt-4, the COM-VEGF-C and LVD had no impact on survival. As a result of multivariate analysis, male, advanced stage (III and IV) and high VD were independent negative prognostic indicators. The positive COM-VEGF also showed weaker correlation with poor survival (Table 4).

### DISCUSSION

Neovascularization has been shown to be necessary for tumour growth and metastasis (Folkman, 1990). Although some contraries exist, many studies have confirmed the negative impact of tumour vascularization on prognosis (Chodak et al, 1980; Weidner et al, 1991; Macchiarini et al, 1992). Among the many reported angiogenic factors, VEGF is the most powerful endothelial cell-specific mitogen associated with tumour neovascularization. A number of investigators have reported a significant relationship between VD and VEGF expression in a variety of tumours (Toi et al, 1994; Mattern et al, 1995, Samoto et al, 1995, Takahashi et al, 1995), and overexpression of VEGF has been associated with poor prognosis in some neoplasms (Toi et al, 1994; Takahashi et al, 1995). However, little information is available on tumour angiogenesis in malignant mesothelioma. Recently, Kumar-Singh et al (1997) reported that VD in MPM tumours was significantly higher than that in non-neoplastic mesothelium. They also reported that the patients with highly vascularized tumours had a significantly shorter survival than patients with poorly vascularized ones (Kumar-Singh et al, 1997). In this study, higher densitometry index of VEGF compared to the mean value of tumours was found in two out of five normal pleural tissues. If pleura is a tissue with relatively high VEGF, this particular condition may suit malignant development. Among VEGFRs, the KDR and flt-4 expression levels tended to be higher in the MPM tumours compared to normal pleural tissues in this study. Although the percentage of VEGF overexpression was not so high, our data confirmed that VEGF expression was significantly associated with VD and the expression of VEGFRs (KDR and flt-1) in MPM tumours. Furthermore, among the 46 MPM patients undergoing standardized resections, high VD was significantly associated with poor survival. A combination factor with VEGF and its receptors (flt-1 and KDR), COM-VEGF, also had a weaker relationship with outcome, yet the value only trends toward significance due to the small sample size.

Most conventional immunohistochemistry methods for the detection of endothelial cells do not distinguish lymphatics from blood vessels. In this study, we tried to assess the LVD within MPM tumours using the enzyme-histochemistry assay for 5′-NA based on the leading method (Wachstein et al, 1954). 5′-NA expression in vascular endothelial cells varies according to vessel types (Turner et al, 1987; Airas et al, 1997). That is, the activity of 5′-NA is very high in lymphatic endothelial cells, while it is very low or absent in blood capillary endothelial cells (Turner et al, 1987). Therefore, this assay has a great potential to discriminate neo-lymphatics from neo-blood vessels after the adequate inhibition of its activity by paraformaldehyde (Vetter et al, 1970; Ji et al, 1997). In normal lymphatics, this enzyme activity appears to be necessary for the growth and development of vessels (Ji et al, 1997), and has an important role in the control of interactions between lymphocytes and vascular endothelial cells (Airas et al, 1997). If this enzyme activity is essential for the proliferation of lymphatic vessels, the same condition may be expected in tumour-induced neo-vessels. Although the significance of 5′-NA activity in cancerous lesions is not clear, this enzyme has been reported in some tumour tissues, including seminoma, malignant fibrous histiocytoma, breast cancer and renal cancer (Wachstein et al, 1954; Wood et al, 1986; Canbolat et al, 1996; Durak et al, 1997). In this study, we have used this method, for the first time, to study the microlymphatic vessel density in MPM tumours and found that 5′-NA staining was limited to vessels in 31 of the 54 (57.4%) MPM tumour specimens. In these samples, lymphatic vessel specificity was optimized by paraformaldehyde fixation for 30–40 min. In 23 cases (42.6%), 5′-NA staining was also noted in MPM tumour cells and within stromal elements. In 20 out of these 23 cases, the intensity of the non-vessel staining was less than that of the vessels, and LVD could be assessed. In three cases with the intensity of non-specific

© 1999 Cancer Research Campaign staining, LVD was assessed by avoiding areas with high non-specific activity. Under the condition that very little information is available about the mechanisms of lymphangiogenesis, some reports have recently suggested that the specific pathways involved in lymphangiogenesis are different from those in hemangiogenesis (Wilting et al, 1996; Oh et al, 1997). Among the VEGF family members, the function of VEGF-C appears to extend to the lymphatic system as a ligand for flt-4, and VEGF-C is suspected to play an important role in lymphangiogenesis (Kukk et al, 1996; Jeltsch et al, 1997; Oh et al, 1997). In this study, we identified a strong positive relationship between VEGF-C mRNA expression and LVD, and flt-4 expression and LVD in MPM, as well as a close association between flt-4 and VEGF-C expression. Further, the mean vessel count for VD that includes both blood and lymphatic vessels was much greater than that for LVD. VD itself had no relationship with VEGF-C and flt-4 expression levels in this study. These results support the use of the enzyme-histochemistry method based on 5′-NA activity for assessment of lymphatics, and also suggest that VEGF-C plays a key role in lymphangiogenesis in MPM. In addtion, flt-4 expression, which has originally been found in lymphatic endothelium (Kaipainen et al, 1995; Joukov et al, 1996), was high in both MPM cell lines used and resected tumour tissues. Immunohistochemical staining revealed flt-4 expression in MPM tumour cells except for some vascular endothelial cells. In this study, it was difficult to clarify the correspondence between endothelial cells with flt-4 expression and those with 5′-NA activity partly owing to the situation that mesothelioma cells themselves expressed both flt-4 and 5′-NA. Although the role of flt-4 in tumour cells is not well known, flt-4 expression was confirmed in mesothelial cells (Hewett et al, 1996). This suggests a possibility that mesothelioma cells express flt-4, and that VEGF-C may have some role for the autocrine growth or proliferation of tumour cells.

In conclusion, our study suggests that VEGF and VEGF-C play an important role in angiogenesis and lymphangiogenesis in MPM tumours, and that VD has a significant impact on the overall survival of MPM patients. The LVD assessed by enzyme-histochemistry for 5′-NA has no impact on nodal metastasis. However, nodal status does not have a relation with outcome in our samples. Therefore, a question remains whether VEGF and VEGF-C have a relation with prognosis in cases where nodal status has a great impact on survival. The potential roles of VEGF and VEGF-C in lymphatic metastasis warrant further study.

### ACKNOWLEDGEMENTS

We would like to thank Mr Larry Tait for technical assistance. We also thank Professor H Yamamoto of Kanazawa University for his kind gift of the primers for flt-1, flt-4 and KDR.

### REFERENCES

Airas L, Niemela J, Salmi M, Puurunen T, Smith DJ and Jalkanen S (1997) Differential regulation and function of CD73, a glycosyl-phosphatidylinositol-linked 70-kD adhesion molecule, on lymphocytes and endothelial cells. *J Cell Biol* **136**: 421–431

Canbolat O, Durak I, Cetin R, Kavutcu M, Demirci S and Ozturk S (1996) Activities of adenosine deaminase, 5′-nucleotidase, guanase, and cystidine deaminase enzymes in cancerous and non-cancerous human breast tissues. *Breast Cancer Res Treat* **37**: 189–193

Chodak GW, Haudenschild C, Gittes RF and Folkman J (1980) Angiogenic activity as a marker of neoplasia and preneoplasia in lesions of the human bladder. *Ann Surg* **192**: 762–771

Durak I, Beduk Y, Kavutcu M, Suzer O, Yaman O, Ozturk HS, Canbolat O and Ulutepe S (1997) Activity of the enzymes participating in purine metabolism of cancerous and non-cancerous human kidney tissues. *Cancer Invest* **5**: 212–216

Folkman J (1990) What is the evidence that tumors are angiogenesis dependent? *J Natl Cancer Inst* **82**: 4–6

Folkman J (1995) Angiogenesis in cancer, vascular, rheumatoid and other disease. *Nat Med* **1**: 27–31

Grimmond S, Lagercrantz J, Drinkwater C, Silins G, Townson S, Pollock P, Gotley D, Carson E, Rakar S, Nordenskjold M, Ward L, Hayward N and Weber G (1996) Cloning and characterization of a novel human gene related to vascular endothelial growth factor. *Genome Res* **6**: 124–131

Hewett PW and Murray JC (1996) Coexpression of flt-1, flt-4 amd KDR in freshly isolated and cultured human endothelial cells. *Biochem Biophys Res Commun* **221**: 697–702

Jeltsch M, Kaipainen A, Joukov V, Meng X, Lakso M, Rauvala H, Swartz M, Fukumura D, Jain RK and Alitalo K (1997) Hyperplasia of lymphatic vessels in VEGF-C transgenic mice. *Science* **276**: 1423–1425

Ji RC and Kato S (1997) Enzyme-histochemical study on postnatal development of rat stomach lymphatic vessels. *Microvasc Res* **54**: 1–12

Joukov V, Pajusola K, Kaipainen A, Chilov D, Lahtinen I, Kukk E, Saksela O, Kalkkinen N and Alitalo K (1996) A novel vascular endothelial growth factor, VEGF-C, is a ligand for the flt-4 (VEGFR-3) and KDR (VEGFR-2) receptor tyrosine kinases. *EMBO J* **15**: 290–298

Kaipainen A, Korhonen J, Mustonen T, van Hinsbergh VWM, Fang G, Dumont D, Breitman M and Alitalo K (1995) Expression of the fms-like tyrosine kinase 4 gene becomes restricted to lymphatic endothelium during development. *Proc Natl Acad Sci USA* **92**: 3566–3570

Kukk E, Lymboussaki A, Taira S, Kaipainen A, Jeltsch M, Joukov V and Alitalo K (1996) VEGF-C receptor binding and pattern of expression with VEGFR-3 suggests a role in lymphatic vascular development. *Development* **122**: 3829–3837

Kumar-Singh S, Vermeulen PB, Weyler J, Segers K, Weyn B, van Daele A, Dirix LY, van Oosterom AT and van Marck E (1997) Evaluation of tumour angiogenesis as a prognostic marker in malignant mesothelioma. *J Pathol* **182**: 211–216

Lee J, Gray A, Yuan J, Luoh S, Avraham H and Wood WI (1996) Vascular endothelial growth factor-related protein: a ligand and specific activator of the tyrosine kinase receptor flt-4. *Proc Natl Acad Sci USA* **93**: 1988–1992

Macchiarini P, Fontanini G, Hardin MJ, Squartini F and Angeletti CA (1992) Relation of neovascularization to metastasis of non-small-cell lung cancer. *Lancet* **340**: 145–146

Mattern J, Koomagi R and Volm M (1995) Vascular endothelial growth factor expression and angiogenesis in non-small-cell lung carcinomas. *Int J Oncol* **6**: 1059–1062

Meyer M, Clauss M, Lepple-Wienhues A, Waltenberger J, Augustin HG, Ziche M, Lanz C, Bittner M, Rziha HJ and Dehio C (1999) A novel vascular endothelial growth factor encoded by Orf virus, VEGF-E, mediates angiogenesis via signalling through VEGFR-2 (KDR) but not VEGFR-1 (Flt-1) receptor tyrosine kinase. *EMBO J* **18**: 363–374

Mossman BT, Kamp DW and Weitzman SA (1996) Mechanisms of carcinogenesis and clinical features of asbestos-associated cancers. *Cancer Invest* **14**: 466–480

Oh SJ, Jeltsch MM, Birkenhager R, McCarthy JE, Weich HA, Christ B, Alitalo K and Wilting J (1997) VEGF and VEGF-C: specific induction of angiogenesis and lymphangiogenesis in the differentiated avian chorioallantoic membrane. *Dev Biol* **188**: 96–109

Olofsson B, Pajusola K, Kaipainen A, von Euler G, Joukov V, Saksela O, Orpana A, Pettersson RF, Alitalo K and Eriksson U (1996) Vascular endothelial growth factor B, a novel growth factor for endothelial cells. *Proc Natl Acad Sci USA* **93**: 2576–2581

Pass HI, Stevens EJ, Oie H, Tsokos MG, Abati AD, Fetsch PA, Mew DJY, Pogrebniak HW and Matthews WJ (1995) Characteristics of nine newly derived mesothelioma cell lines. *Ann Thorac Surg* **59**: 835–8344

Rusch VW (1996) A proposed new International TNM staging system for malignant pleural mesothelioma from the International Mesothelioma Interest Group. *Lung Cancer* **14**: 1–12

Samoto K, Ikezaki K, Ono M, Shono T, Kohno K, Kuwano M and Fukui M (1995) Expression of vascular endothelial growth factor and its possible relation with neovascularization in human brain tumor. *Cancer Res* **55**: 1189–1193

Takahashi Y, Kitadai Y, Bucana CD, Cleary KR and Ellis LM (1995) Expression of vascular endothelial growth factor and its receptor, KDR, correlates with vascularity, metastasis, and proliferation of human colon cancer. *Cancer Res* **55**: 3964–3968

Toi M, Hoshina S, Takayanagi T and Tominaga T (1994) Association of vascular endothelial growth factor expression with tumor angiogenesis and with early relapse in primary breast cancer. *Jpn J Cancer Res* **85**: 1045–1049

© 1999 Cancer Research Campaign

Turner RR, Beckstead JH, Warnke RA and Wood GS (1987) Endothelial cell phenotypic diversity. In situ demonstration of immunologic and enzymatic heterogeneity that correlates with specific morphologic subtypes. *Am J Clin Pathol* **87**: 569–575

Vetter W (1970) Alkaline phosphatasen in mastzellen, blut- und lymphagefäβen der rattenzunge. 5′-nucleotidase-, unspezifische alkalische phosphatase- und polyphosphatase- (ATP′ase) aktivität unter besondere berückscichtigung des pH. *Z Anat Entwicklungsgesch* **130**: 153–176

Wachstein M and Meisel E (1954) The histochemical distribution of 5-nucleotidase and unspecific alkaline phosphatase in the testicle of various species and in two human seminomas. *J Histochem* **2**: 137–148

Weidner N, Semple JP, Welch WR and Folkman J (1991) Tumor angiogenesis and metastasis – correlation in invasive breast carcinoma. *N Engl J Med* **324**: 1–8

Wilting J, Birkenhager R, Eichmann A, Kurz H, Martiny-Baron G, Marme D, McCarthy JE, Christ B and Weich HA (1996) VEGF121 induces proliferation of vascular endothelial cells and expression of flk-1 without affecting lymphatic vessels of chorioallantoic membrane. *Dev Biol* **176**: 76–85

Wood GS, Beckstead JH, Turner RR, Hendrickson MR, Kempson RL and Warnke RA (1986) Malignant fibrous histiocytoma tumor cells resemble fibroblasts. *Am J Surg Pathol* **110**: 323–335

Yamada Y, Nezu J, Shimane M and Hirata Y (1997) Molecular cloning of a novel vascular endothelial growth factor, VEGF-D. *Genomics* **42**: 483–488

*© 1999 Cancer Research Campaign*
